# Influenza clinical testing and oseltamivir treatment in hospitalized children with acute respiratory illness, 2015–2016

**DOI:** 10.1111/irv.12927

**Published:** 2021-10-26

**Authors:** Lubna Hamdan, Varvara Probst, Zaid Haddadin, Herdi Rahman, Andrew J. Spieker, Simon Vandekar, Laura S. Stewart, John V. Williams, Julie A. Boom, Flor Munoz, Janet A. Englund, Rangaraj Selvarangan, Mary A. Staat, Geoffrey A. Weinberg, Parvin H. Azimi, Eileen J. Klein, Monica McNeal, Leila C. Sahni, Monica N. Singer, Peter G. Szilagyi, Christopher J. Harrison, Manish Patel, Angela P. Campbell, Natasha B. Halasa

**Affiliations:** ^1^ Department of Pediatrics, Division of Infectious Diseases Vanderbilt University Medical Center Nashville Tennessee USA; ^2^ Department of Biostatistics Vanderbilt University Medical Center Nashville Tennessee USA; ^3^ Pediatric Infectious Diseases, Institute for Infection, Inflammation, and Immunity in Children, University of Pittsburgh School of Medicine UPMC Children's Hospital of Pittsburgh Pittsburgh Pennsylvania USA; ^4^ Primary Care Practice at Palm Center, Immunization Project, Baylor College of Medicine Texas Children's Hospital Houston Texas USA; ^5^ Pediatrics and Molecular Virology and Microbiology, Baylor College of Medicine Texas Children's Hospital Houston Texas USA; ^6^ Department of Pediatrics, Division of Infectious Diseases Seattle Children's Hospital Seattle Washington USA; ^7^ Pathology & Laboratory Medicine Children's Mercy Hospital Kansas City Missouri USA; ^8^ Pediatric Infectious Diseases, University of Cincinnati College of Medicine Cincinnati Children's Hospital and Medical Center Cincinnati Ohio USA; ^9^ Pediatric Infectious Diseases University of Rochester Medical Center Rochester New York USA; ^10^ Pediatric Infectious Diseases Children's Hospital and Research Center Oakland California USA; ^11^ Department of Pediatrics, Division of Emergency Medicine Seattle Children's Hospital Seattle Washington USA; ^12^ Department of Pediatrics, Section of Hematology‐Oncology, Baylor College of Medicine Texas Children's Hospital Houston Texas USA; ^13^ Department of Pediatrics University of California at Los Angeles Mattel Children's Hospital Los Angeles California USA; ^14^ Pediatric Infectious Diseases Children's Mercy Hospital Kansas City Missouri USA; ^15^ National Center for Immunization and Respiratory Diseases, Division of Viral Diseases Centers for Disease Control and Prevention Atlanta Georgia USA; ^16^ Epidemiology and Prevention Branch, Influenza Division, National Center for Immunization and Respiratory Diseases Centers for Disease Control and Prevention Atlanta Georgia USA

**Keywords:** clinical testing, hospitalization, influenza, nucleic acid amplification tests (NAAT), oseltamivir, rapid influenza testing

## Abstract

**Background:**

Antiviral treatment is recommended for all hospitalized children with suspected or confirmed influenza, regardless of their risk profile. Few data exist on adherence to these recommendations, so we sought to determine factors associated with influenza testing and antiviral treatment in children.

**Methods:**

Hospitalized children <18 years of age with acute respiratory illness (ARI) were enrolled through active surveillance at pediatric medical centers in seven cities between 11/1/2015 and 6/30/2016; clinical information was obtained from parent interview and chart review. We used generalized linear mixed‐effects models to identify factors associated with influenza testing and antiviral treatment.

**Results:**

Of the 2299 hospitalized children with ARI enrolled during one influenza season, 51% (*n* = 1183) were tested clinically for influenza. Clinicians provided antiviral treatment for 61 of 117 (52%) patients with a positive influenza test versus 66 of 1066 (6%) with a negative or unknown test result. In multivariable analyses, factors associated with testing included neuromuscular disease (aOR = 5.35, 95% CI [3.58–8.01]), immunocompromised status (aOR = 2.88, 95% CI [1.66–5.01]), age (aOR = 0.93, 95% CI [0.91–0.96]), private only versus public only insurance (aOR = 0.78, 95% CI [0.63–0.98]), and chronic lung disease (aOR = 0.64, 95% CI [0.51–0.81]). Factors associated with antiviral treatment included neuromuscular disease (aOR = 1.86, 95% CI [1.04, 3.31]), immunocompromised state (aOR = 2.63, 95% CI [1.38, 4.99]), duration of illness (aOR = 0.92, 95% CI [0.84, 0.99]), and chronic lung disease (aOR = 0.60, 95% CI [0.38, 0.95]).

**Conclusion:**

Approximately half of children hospitalized with influenza during the 2015–2016 influenza season were treated with antivirals. Because antiviral treatment for influenza is associated with better health outcomes, further studies of subsequent seasons would help evaluate current use of antivirals among children and better understand barriers for treatment.

## 1 INTRODUCTION

Influenza is an important cause of acute respiratory illnesses (ARI), with an estimated 4.3–21 million clinical outpatient visits, 140,000–810,000 hospitalizations, and 12,000–61,000 deaths annually over the past decade in the United States (U.S.).^1^, ^2^, ^3^, ^4^ Influenza has a high attack rate in children with an estimated incidence of 19/1000 per year and an overall mortality rate of 15 deaths per 1000 influenza‐positive children.^5^ Children less than 5 years of age, and especially children less than 2 years of age, American Indians or Alaska Natives and those with underlying comorbidities are at higher risk for developing influenza‐associated complications.^6^ However, nearly 50% of hospitalized children with influenza do not have an underlying medical condition.^7^

Influenza vaccination is the mainstay of prevention against influenza disease and can prevent influenza‐associated complications in children 6 months of age and older.^8^ If an infection is acquired, antiviral treatment for influenza disease has been shown to reduce complications, shorten the length of hospitalization, and reduce mortality; these benefits are more pronounced when treatment is initiated within 48 h of symptom onset.^9^, ^10^, ^11^ However, variation in the prescribing patterns among clinicians exist, possibly due to concerns about effectiveness and reporting biases in industry funded trials.^12^, ^13^ Detailed recommendations by the Infectious Diseases Society of America (IDSA) for antiviral treatment for influenza disease were published in response to the 2009 pandemic and has been updated periodically.^14^ The recommendations include antiviral treatment for all hospitalized individuals with confirmed or suspected influenza, regardless of underlying illness or vaccination status, and recommend initiation of treatment within 48 h of symptom onset.^14^ In addition, the recommendations advise that persons hospitalized for confirmed influenza may also benefit from treatment even if initiated more than 48 h after the onset of illness.^14^ The American Academy of Pediatrics (AAP) recommends treatment as soon as possible for children hospitalized with suspected influenza, hospitalized for severe, complicated, or progressive illness attributable to influenza regardless of duration of symptoms, and to children with suspected influenza and at increased risk of complications. It also recommends considering treatment for any healthy child with suspected influenza, and to healthy children with suspected influenza who live with a household contact who is <6 months old or has a medical condition that predisposes them to complications.^15^ Despite these recommendations, controversy among physicians exists and suboptimal antiviral use has been reported in recent studies.^16^, ^17^

Clinical testing for influenza illness may influence antiviral treatment; however, based on updated 2018 IDSA recommendations, antiviral treatment decisions should not be delayed until laboratory confirmation of influenza.^6^ Data are limited on factors associated with making clinical decisions whether to test for and/or to treat influenza. This study describes influenza testing and antiviral treatment practices in children admitted with a diagnosis of ARI during the 2015–2016 influenza season and identifies factors associated with decisions for influenza testing and antiviral treatment.

## 2 METHODS

### 2.1 Study description

As part of the Centers for Disease Control and Prevention (CDC) New Vaccine Surveillance Network (NVSN), active population‐based ARI surveillance was conducted from November 1, 2015, through June 30, 2016, at pediatric medical centers in seven cities: Rochester, New York; Cincinnati, Ohio; Nashville, Tennessee; Kansas City, Missouri; Houston, Texas; Seattle, Washington; and Oakland, California.^18^

### 2.2 Study population

Children <18 years old were eligible for enrollment if they were admitted to a participating hospital, resided within each hospital's surveillance area, had illness duration <14 days, were enrolled in the study within 48 h of admission, and had one or more of the following admission diagnoses: acute respiratory illness, apnea, asthma exacerbation, reactive airways disease, bronchiolitis, croup, cystic fibrosis exacerbation, respiratory syncytial virus infection, febrile neonate, febrile seizure, hypothermia, paroxysmal cough, wheezing, influenza, fever without localizing signs, respiratory distress, otitis media, pharyngitis, pneumonia, pneumonitis, rule‐out sepsis, sinusitis, tonsillitis, streptococcal pharyngitis, upper respiratory infection, other respiratory infection, bronchiectasis, tracheitis, and/or pleural effusion.^18^

Children were excluded if they had a known nonrespiratory cause for hospitalization, had fever and neutropenia with malignancy, were discharged from a hospital in the prior 4 days, were transferred after admission at another hospital for 48 h, had never been discharged home after birth, or had previously enrolled in this study <14 days prior to their current admission.^18^

**FIGURE 1:**
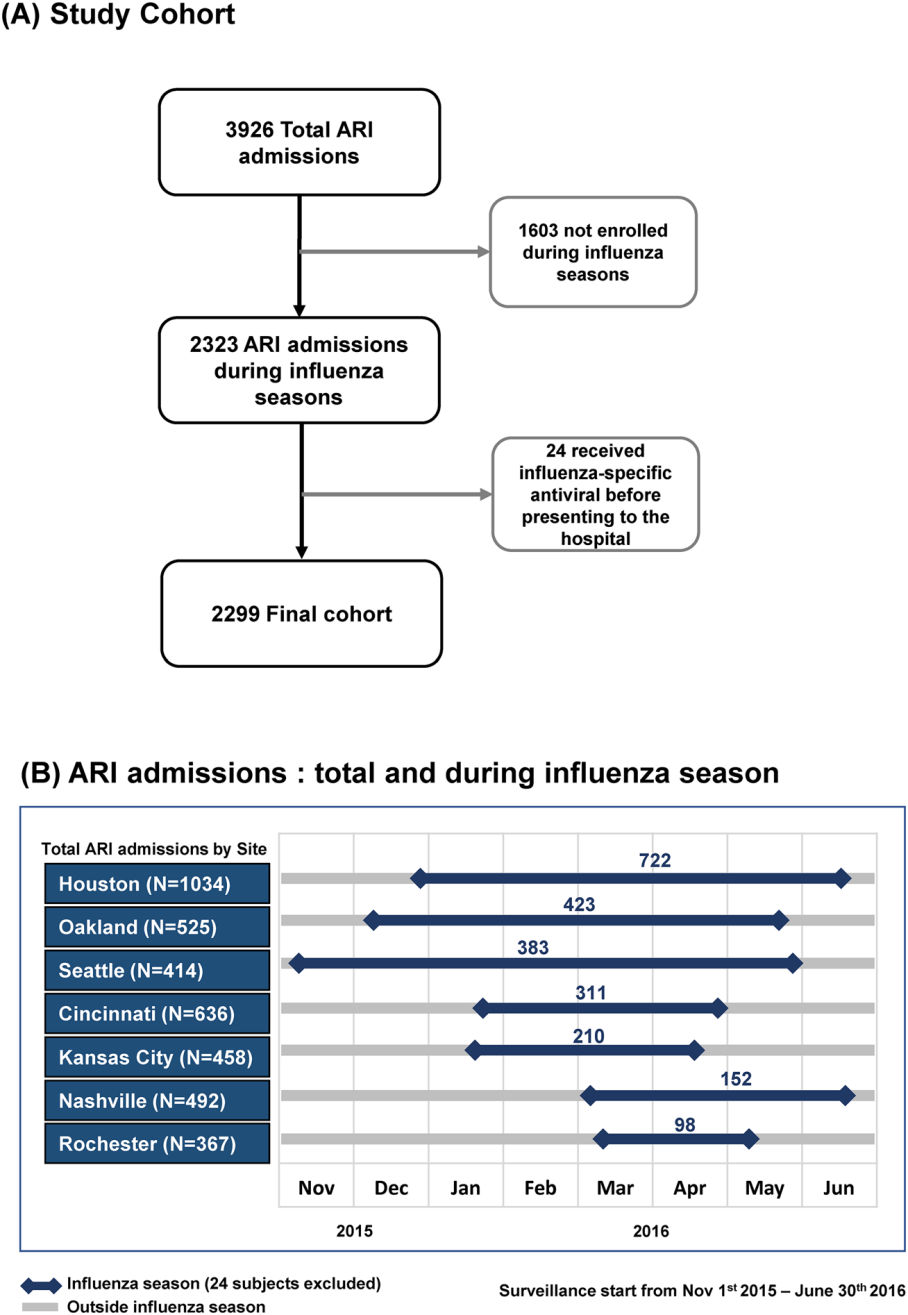
(A) Study cohort including total admissions due to acute respiratory illnesses and excluded subjects. (B) Acute respiratory illness admissions: total and during influenza season

For this study, we included children who were hospitalized during each site's influenza season defined as the date of first through the last influenza positive case for each site based on research testing results (Figure 1A). We excluded 24 children who received influenza antiviral treatment prior to hospitalization (Figure 1A).

### 2.3 Study design

Following written informed consent from a parent or guardian and assent when applicable, demographic data, history of current illness, social history and treatment received before presentation were collected through parent/guardian interviews. Standardized medical chart reviews were performed, and clinical interventions and outcome data were collected including chronic comorbid conditions, types and results of clinical influenza diagnostic studies performed, antiviral treatment, intensive care unit (ICU) admissions, and oxygen requirement.

Institutional Review Board approval was obtained from the CDC and at each individual site.

### 2.4 Definitions

#### 2.4.1 Influenza season

Influenza season was defined as the period between the dates of the first through last influenza positive case for each specific site, based upon research laboratory testing. Research laboratory diagnostic influenza assays varied by site, but all were nucleic acid amplification tests (NAATs) for which CDC‐generated influenza proficiency panels were successfully completed.^19^

Influenza clinical testing was defined as any influenza testing that was ordered by providers. Clinical testing was available for those subjects whose treating provider ordered testing from the clinical laboratory of their respective hospital as part of standard care; the method of clinical laboratory testing was either rapid influenza diagnostic testing (RIDT) or NAATs. Positive test results were defined according to results documented in medical charts for influenza A, influenza A/(H1N1)pdm09, influenza A(H3N2), and influenza B lineage viruses.

#### 2.4.2 Antiviral use

Influenza antiviral use was defined as in‐hospital receipt of a neuraminidase inhibitor (oseltamivir or zanamivir) or adamantane (amantadine or rimantadine) documented by chart review.

#### 2.4.3 Underlying medical condition

Underlying medical conditions included chronic pulmonary/airway, cardiac, gastrointestinal, liver, kidney, endocrine, neurologic/neuromuscular, hematologic/oncologic, genetic/metabolic, or immunocompromised conditions. Chronic lung disease included asthma, cystic fibrosis, bronchopulmonary dysplasia, and other lung disorders recorded in the chart. Neuromuscular disease included cerebral palsy, seizures, and other neuromuscular diseases recorded in the chart. Immunocompromised included children with a history of immunodeficiency/immunosuppressive condition, transplant (peripheral blood stem cells, bone marrow, cord blood, or solid organ), cancer diagnosis within the prior 5 years, and sickle cell anemia.

#### 2.4.4 Influenza vaccine reporting

Receipt of influenza vaccine was determined by parental report of receiving influenza vaccination for the current season for children who were 6 months or older.

### 2.5 Data analysis

All analyses were performed using Stata IC 15.0 (StatCorp LLC, College Station, TX) or R version 4.0.3. Data were collected and managed the CDC's Secure Access Management Services (SAMS).

Demographic and clinical characteristics were evaluated using descriptive statistics (frequency and percentage for categorical variables, or mean and standard deviation for continuous variables). Between‐group comparisons were performed using Pearson's chi‐squared test for categorical variables and two‐sample *t* tests of mean differences for continuous variables.

We used a generalized linear mixed‐effects model on the log‐odds scale to evaluate factors associated with influenza testing and antiviral treatment, separately.^20^ To address missing data, we used multiple imputation via chained equations with *M* = 500 iterations, aggregating results using Rubin's rules.^21^, ^22^ The following predictors were included a priori in each of the two models: continuous age (years), sex, race/ethnicity (non‐Hispanic White, non‐Hispanic Black, Hispanic, and other), fever, cough, fever & cough, duration of illness prior to admission, chronic lung disease, neuromuscular disease, immunocompromised status, congenital heart disease, influenza vaccination, and insurance status (public, private, both, and self‐pay).^2^, ^12^, ^23^, ^24^ We included a random intercept for each study site. From these models, we estimated adjusted odds ratios for each predictor and derived corresponding Wald‐based 95% confidence intervals and p‐values. Statistical significance was determined at the nominal α = 0.05 level (two‐sided).

## 3 RESULTS

### 3.1 Patient characteristics

Among the 3926 enrolled children who were hospitalized with ARI or febrile illness between July 2015 and June 2016, 2299 (58%) met eligibility criteria for this analysis (Figure 1A). The total duration of influenza season by each site is represented in Figure 1B. The Houston and Oakland sites had the highest numbers of enrollments representing half of the cohort, and Seattle had the longest influenza season (Figure 1B).

For children who were enrolled and eligible, the mean age was 2.8 years (median 1 year, IQR [0–4]), 56% were under 2 years, 58% male, and 44% had at least one underlying medical condition. The mean duration from symptom onset to admission was 3.3 days; 43% had symptoms for ≤2 days prior to hospitalization.

### 3.2 Clinical influenza testing

Among our population, 1183 (51%) were tested for influenza (Table 1): 24% RIDT, 71% NAATs, and 6% both. Factors with significant positive association with testing included neuromuscular disease (aOR = 5.35, 95% CI [3.58–8.01]), congenital heart disease (aOR = 2.52, 95% CI [1.59–3.99]), and immunocompromised status (aOR = 2.88, 95% CI [1.66–5.01]), while those negatively associated with testing included age (aOR = 0.93, 95% CI [0.91–0.96]), private vs. public insurance (aOR = 0.78, 95% CI [0.63–0.98]) and chronic lung disease (aOR = 0.64, 95% CI [0.51–0.81]) (Table 2).

**TABLE 1:** Demographics and clinical characteristics of children hospitalized with ARI during 2015–2016 influenza season

	Treated 149/2299 (6.5%) *n* (%) Mean ± SD Median [IQR]	Tested 1183/2299 (51.5%) *n* (%) Mean ± SD Median [IQR]
Demographics		
Age (years), mean	3.2 ± 4.2	2.4 ± 3.6
Age (years), median	1 [0–5]	1 [0–3]
Sex, Male	92/1337 (6.9%)	680/1337 (50.9%)
Race		
Non‐Hispanic White	34/735 (4.6%)	377/735 (51.3%)
Non‐Hispanic Black	37/478 (7.7%)	198/478 (41.4%)
Hispanic	69/798 (8.7%)	448/798 (56.14%)
Other	7/274 (2.6%)	152/274 (55.5%)
Insurance status		
Private	35/746 (4.7%)	35/746 (4.7%)
Public	107/1375 (7.8%)	107/1375 (7.8%)
Both	4/112 (3.6%)	4/112 (3.6%)
Self‐pay	2/59 (3.4%)	2/59 (3.4%)
Clinical characteristics		
Chronic lung diseases	33/690 (4.9%)	287/690 (41.6%)
Neuromuscular diseases	21/201 (14.1%)	160/201 (79.6%)
Congenital heart disease	11/115 (9.6%)	84/115 (73%)
Immunocompromised	16/80 (20%)	59/80 (73.8%)
Influenza vaccination ≥6 months	68/1085 (6.3%)	555/1085 (51.2%)
Antiviral treatment	149/149 (100%)	127/149 (85.2%)
Clinical testing for influenza	127/1183 (10.7%)	1183/1183 (100%)
Type of test		
Rapid	19/279 (6.8%)	279/279 (100%)
Molecular	100/834 (12%)	834/834 (100%)
Both	8/70 (11.4%)	70/70 (100%)
Clinical outcome		
Required oxygen support	87 (6.4%)	758 (55.6%)
ICU admission	38/378 (10.1%)	286 (75.7%)
Intubated	13/72 (18.1%)	59/72 (81.9%)
Length of hospitalization (days), mean	4 ± 5.7	3.9 ± 6.5
Length of hospitalization (days), median	2 [2–4]	2 [1–4]

**TABLE 2:** Generalized linear mixed‐effects model evaluating predictors of testing

Variable	Odds ratio	95% CI	*p* value
Age (years)	0.93	[0.91, 0.96]	**<0.001**
Sex, male	0.90	[0.75, 1.09]	0.28
Race/ethnicity: non‐Hispanic White	*REF*	*REF*	*REF*
Race/ethnicity: non‐Hispanic Black	0.86	[0.65, 1.14]	0.30
Race/ethnicity: Hispanic	1.08	[0.82, 1.41]	0.59
Race/ethnicity: other	1.08	[0.78, 1.48]	0.66
Fever	1.94	[0.97, 3.88]	0.062
Cough	1.06	[0.58, 1.94]	0.85
Fever and cough	0.81	[0.40, 1.68]	0.58
Duration of illness (days)	0.97	[0.93, 1.01]	0.18
Chronic lung disease	0.64	[0.51, 0.81]	**<0.001**
Neuromuscular disease	5.35	[3.58, 8.01]	**<0.001**
Immunocompromised	2.88	[1.66, 5.01]	**<0.001**
Congenital heart disease	2.52	[1.59, 3.99]	**<0.001**
Influenza vaccination ≥6 months	1.09	[0.86, 1.38]	0.48
Insurance: public	*REF*	*REF*	*REF*
Insurance: private	0.78	[0.63, 0.98]	**0.030**
Insurance: public and private	0.96	[0.62, 1.49]	0.86
Insurance: self‐pay	1.01	[0.58, 1.76]	0.98
Peak months	0.84	[0.69, 1.02]	0.073

*Note*: Bold values denote statistical significance.

Of the 1183 tested, 117 (10%) were influenza positive. Compared to influenza‐negative children, children with influenza were older (mean age, 3.6 vs. 2.3 years), more likely to have neuromuscular disease (14% vs. 4%), have congenital heart disease (7% vs. 3%), be immunocompromised (5% vs. 2%), receive oseltamivir (11% vs. 2%), require oxygen support (65% vs. 55%), be admitted to the ICU (24% vs. 8%), and be intubated (5% vs. 1%), all *p* < 0.001. Influenza positive patients were less likely than influenza negative patients to have chronic lung disease (24% vs. 36%, *p* < 0.001).

### 3.3 Antiviral treatment

All treated patients received oseltamivir. Antiviral treatment was positively associated with neuromuscular disease (aOR = 1.86, 95% CI [1.04, 3.31]), and immunocompromised state (aOR = 2.63, 95% CI [1.38, 4.99]) and was negatively associated with duration of illness (aOR = 0.92, 95% CI [0.84, 0.99]) and chronic lung disease (aOR = 0.60, 95% CI [0.38, 0.95]) (Table 3).

**TABLE 3:** Generalized linear mixed‐effects model evaluating predictors of treatment

Variable	Odds ratio	95% CI	*p* value
Age (years)	1.04	[0.99, 1.09]	0.17
Sex, male	1.19	[0.83, 1.70]	0.34
Race/ethnicity: non‐Hispanic White	*REF*	*REF*	*REF*
Race/ethnicity: non‐Hispanic Black	1.41	[0.81, 2.45]	0.22
Race/ethnicity: Hispanic	1.38	[0.83, 2.29]	0.22
Race/ethnicity: other	0.73	[0.31, 1.70]	0.46
Fever	5.01	[0.64, 39.4]	0.13
Cough	2.19	[0.29, 16.8]	0.45
Fever and cough	0.68	[0.08, 5.64]	0.72
Duration of illness	0.92	[0.84, 0.99]	**0.035**
Chronic lung disease	0.60	[0.38, 0.95]	**0.028**
Neuromuscular disease	1.86	[1.04, 3.31]	**0.035**
Immunocompromised	2.63	[1.38, 4.99]	**0.003**
Congenital heart disease	1.31	[0.65, 2.61]	0.45
Influenza vaccination ≥6 months	0.82	[0.51, 1.31]	0.41
Insurance: public	*REF*	*REF*	*REF*
Insurance: private	0.74	[0.47, 1.16]	0.20
Insurance: public and private	0.53	[0.18, 1.52]	0.24
Insurance: self‐pay	0.51	[0.12, 2.20]	0.37
Peak months	0.76	[0.51, 1.13]	0.17

*Note*: Bold values denote statistical significance.

Moreover, children who were tested for influenza were more likely to receive antiviral treatment (tested vs. not tested: 127/1183 (11%) vs. 22/1183 (2%), *p* < 0.001). Additionally, children who tested positive were more likely to be treated (positive vs. nonpositive 61/117 (52%) vs. 66/1066 (6%), *p* < 0.001).

## 4 DISCUSSION

In our study of children hospitalized with ARI during the 2015–2016 influenza season across multiple pediatric medical centers, influenza testing during the influenza season was infrequent and antiviral treatment among influenza positive children was low. Additionally, only half of the children with laboratory‐confirmed influenza during standard care received antiviral treatment despite the IDSA and AAP recommendations of empiric antiviral treatment for hospitalized patients with confirmed or suspected influenza without the need for testing.

Shorter duration of illness was associated with higher odds of antiviral treatment. The 2009 IDSA guidelines recommended empiric antiviral treatment for hospitalized patients with confirmed or suspected influenza if treatment can be commenced within 48 h of symptom onset.^14^ The updated guidelines in 2018 included antiviral treatment for all hospitalized individuals with confirmed or suspected influenza, regardless of the illness duration prior to hospitalization.^6^ Supportive evidence mainly depended on adult studies. Among the few studies investigating antiviral effects in children, early treatment was found to shorten the duration of symptoms, decrease hospital admissions, and reduce the risk for developing otitis media.^25^, ^26^ In hospitalized children, early antiviral treatment with oseltamivir also shortened the duration of hospitalization.^9^ Therefore, further investigation to understand barriers for antiviral treatment and physicians' perceptions of antiviral may be useful.

Suboptimal antiviral use among hospitalized patients has been reported previously, especially in the pre‐H1N1‐2009 pandemic era. A prior NVSN study with three clinical sites between 2004 and 2009 reported that only 1.5% of hospitalized children under 5 years of age with research‐confirmed influenza received antiviral treatment.^2^ Other data from 2003 to 2008 from children ≥1 year who had a positive clinical influenza test within 48 h of symptom onset, 37%–48% were treated with an antiviral.^24^ In contrast, during the 2009‐H1N1 pandemic, a study noted that 77% of children hospitalized with influenza received an antiviral but a 27% decline was reported the following year.^17^ The FluSurv‐NET subsequently reported that 72% of 6469 hospitalized children with confirmed influenza received antivirals between 2010 and 2015.^16^ Only evaluating populations with positive clinical testing compared to broader populations that might be eligible for antiviral treatment may overestimate the antiviral coverage.^27^ Our findings indicate that additional efforts are needed to increase awareness of antiviral effectiveness and current empiric influenza treatment recommendations in hospitalized children with suspected influenza without delay for testing results.

Approximately half of the enrolled children had a provider‐initiated influenza test with the majority having a NAAT performed, which is the recommended test for this population.^28^ Also our study showed an association between testing, testing results, and receiving treatment. Historically, RIDT was the most commonly used diagnostic test.^2^, ^24^, ^29^ While RIDT sensitivity is higher in children than adults, sensitivity in children is estimated to be 67%.^30^ During the 2009 H1N1 pandemic, RIDT was associated with a higher false negative rate and clinicians were directed to start antiviral treatment if influenza was highly suspected despite a negative result.^14^ NAAT for influenza is more sensitive compared to RIDT and is currently more widely used in point of care testing.^30^, ^31^ Although testing for influenza is not required to initiate antiviral treatment, availability of accurate and timely diagnostic tests represents a potential challenge to antiviral use. One study in adults hospitalized with influenza found that 26% of those testing positive for influenza by a provider were treated compared to 5% of those testing negative and to less than 1% of those not tested; 24% of those treated with antivirals were not tested.^27^ Despite the limited sensitivity of RIDT, a study among children seeking care in the Emergency Department showed that a positive RIDT was associated with increased antiviral use.^32^ A recent study documented use of rapid influenza NAAT in acute care settings improved appropriate antiviral treatment decisions in children^33^More studies are needed to evaluate the effect of test methodology on treatment decisions and to confirm potential improved adherence to treatment recommendations since introduction of the NAAT to hospitals.

Strengths of this study include the large pediatric sample size, multi‐center involvement over a large geographic area, and prospective enrollment and collection of data. Our study also has some important limitations. These data are from 2015 to 2016 and only a single influenza season, so additional years are needed to determine the frequency of antiviral treatment and testing for influenza over multiple seasons. New NAAT assays are now more readily available and their faster turnaround times may affect antiviral treatment and testing decisions. We used parental reporting for influenza vaccine history because this information would be readily available to clinicians. Although parental reports of immunization were found to be a reliable predictor of immunization documented in the medical record in some studies, the frequencies may be optimistic given that they were found to be lower if parental report is subsequently subjected to hard‐copy verification.^19^, ^34^, ^35^ Lastly, our sites were major university‐affiliated hospitals and may not represent the practice in all hospitals that care for children.

In summary, antiviral treatment continued to be suboptimal in 2015–2016 in hospitalized children with ARI or febrile illnesses, including those with clinically proven influenza, despite recommendations to treat hospitalized children with confirmed or suspected influenza regardless of symptom duration. Identification of high‐risk groups in addition to testing seemed to positively affect treatment frequencies. Further studies may provide a better understanding of barriers to antiviral treatment among hospitalized children and promote increased use of antivirals for hospitalized children with suspected or confirmed influenza infection.

## AUTHOR CONTRIBUTIONS

**Lubna Hamdan:** Conceptualization; data curation; formal analysis; methodology; visualization. **Varvara Probst:** Data curation; formal analysis; investigation; methodology. **Zaid Haddadin:** Formal analysis; investigation; methodology. **Herdi Rahman:** Data curation; formal analysis; software; validation; visualization. **Andrew Spieker:** Conceptualization; data curation; formal analysis; software; supervision; validation. **Simon Vandekar:** Conceptualization; data curation; formal analysis; software; supervision; validation. **Laura Stewart:** Conceptualization; methodology; project administration; resources; validation. **John Williams:** Conceptualization; investigation; methodology; resources. **Julie Boom:** Conceptualization; investigation; methodology; resources. **Flor Munoz:** Conceptualization; investigation; methodology; resources. **Janet Englund:** Conceptualization; investigation; methodology; resources. **Rangaraj Selvarangan:** Conceptualization; investigation; methodology; validation. **Mary Allen Staat:** Conceptualization; investigation; methodology; resources. **Geoffrey Weinberg:** Conceptualization; investigation; methodology; resources. **Parvin Azimi:** Conceptualization; investigation; methodology; resources. **Eileen Klein:** Conceptualization; investigation; methodology; resources. **Monica Mcneal:** Conceptualization; investigation; methodology; resources. **Laila Sahni:** Conceptualization; investigation; project administration; resources. **Monica Singer:** Conceptualization; investigation; methodology; resources. **Peter Szilagyi:** Conceptualization; investigation; methodology; resources. **Christopher Harrison:** Conceptualization; investigation; methodology; resources. **Manish Patel:** Conceptualization; data curation; investigation; methodology; project administration; resources; supervision; validation. **Angela Campbell:** Conceptualization; investigation; methodology; project administration; resources; supervision; validation. **Natasha B. Halasa:** Conceptualization; funding acquisition; investigation; methodology; resources; supervision; validation; visualization.

### PEER REVIEW

The peer review history for this article is available at https://publons.com/publon/10.1111/irv.12927.

## Data Availability

Unidentified Datasets analyzed during this study are available through the New Vaccine Surveillance Network, CDC
